# Processing of Flavor-Enhanced Oils: Optimization and Validation of Multiple Headspace Solid-Phase Microextraction-Arrow to Quantify Pyrazines in the Oils

**DOI:** 10.3390/life11050390

**Published:** 2021-04-26

**Authors:** Ziyan Xu, Chuan Zhou, Haiming Shi, Hong Zhang, Yanlan Bi, Xuebing Xu

**Affiliations:** 1 College of Food Science and Technology, Henan University of Technology, Lianhua Road, Zhengzhou 450001, China; zyxhghy@163.com (Z.X.); bylzry@126.com (Y.B.); 2 Wilmar (Shanghai) Biotechnology Research and Development Center Co., Ltd., 118 Gaodong Road, Pudong New District, Shanghai 200137, China; zhouchuan@cn.wilmar-intl.com (C.Z.); shihaiming@cn.wilmar-intl.com (H.S.); zhanghongsh@cn.wilmar-intl.com (H.Z.)

**Keywords:** solid-phase microextraction-arrow, multiple headspace solid-phase microextraction, pyrazine, flavor edible oil, internal standard method

## Abstract

An efficient and effective multiple headspace-solid phase microextraction-arrow-gas chromatography-mass spectrometry (MHS-SPME-arrow-GCMS) analytical protocol is established and used to quantify the flavor compounds in oils. SPME conditions, such as fiber coating, pre-incubation temperature, extraction temperature, and time were studied. The feasibility was compared between SPME-arrow and the traditional fiber by loading different sample amounts. It was found that the SPME-arrow was more suitable for the MHS-SPME. The limit of detection (LODs) and limit of quantitation (LOQs) of pyrazines were in the range of 2–60 ng and 6–180 ng/g oil, respectively. The relative standard deviation (RSD) of both intra- and inter-day were lower than 16%. The mean recoveries for spiked pyrazines in rapeseed oil were in the range of 91.6–109.2%. Furthermore, this newly established method of MHS-SPME-arrow was compared with stable isotopes dilution analysis (SIDA) by using [^2^H_6_]-2-methyl-pyrazine. The results are comparable and indicate this method can be used for edible oil flavor analysis.

## 1. Introduction

Good natural flavor is one of the major drivers for consumers to purchase edible oils, especially in Asia. Flavor-enhanced edible oils are normally produced by high roasting temperature in mechanical pressing. Under high temperatures in the processing, a lot of flavor volatiles are created through various reactions, in particular the Mallard reactions. Pyrazines are a group of such volatiles that attract the most importance. A lot of studies have shown that pyrazines are formed by roasting, baking, or thermally processing through Maillard reactions and impart cocoa, peanut, roasted nut-like flavors to various foods [1,2,3].

Generally, pyrazines, which exhibit high odor activity values, are highly correlated with roasted flavor in roasted black tea, coffee, and oils [4,5,6,7]. For instance, the total pyrazine level was closely related to the roasted peanut flavor in peanut by sensory evaluation and correlation analysis [8]. Meanwhile, it was found that 2,5-dimethyl-pyrazine was one of the key flavor compounds for sesame oil (Odor Activity Value, OAV > 100) [6], while 2-ethyl-5(or 6)-methyl-pyrazine, 2,6-diethyl-pyrazine, and 3-ethyl-2,5-dimethyl-pyrazine were the major aroma contributors in the roasted sesame oil [9].

The above pyrazines were also found to be aroma-active compounds in flavor-enhanced rapeseed oils and roasted pumpkin seed oils [10,11]. Interestingly, almost the same group of pyrazines was found in various oils, and variation in the concentration of individual compounds made their flavor different from one to another. This is in line with Poehlmann’s notion that impressions of different flavors of roasted oils are caused by differences in the quantity rather than the quality of their key aroma-active compounds [10].

The conventional methods of extracting the aroma compounds of oils mainly include headspace solid-phase microextraction (HS-SPME), solvent-assisted flavor evaporation (SAFE), simultaneous distillation extraction (SDE), dynamic headspace (DHS), static headspace (SHS), and stir bar sorptive extraction (SBSE) [12,13,14,15]. Compared to other methods, HS-SPME is a simple and easy technique, which reduces the size of extraction instrumentation and operates solvent-free [16], and has been widely used for volatiles analysis in food products, such as pasta [17], bread [18], and edible oils [19].

SPME-arrow fiber is a new device, which has 6–20 times larger than the sorbent phase of traditional SPME fiber and shows more than 10 times the sensitivities and extraction efficacies [20]. Until now, SPME-arrow fiber has been employed for the determination of volatiles of various food products, such as brown rice vinegar [21], Korean salt–fermented fish sauce [20], soy sauce [22], and milk [23]. In these studies, SPME-arrow showed higher extraction performance, such as more kinds of compounds extracted, larger extraction amount, and better reproducibility. Moreover, SPME-arrow was evaluated as more effective in extracting pyrazines in food matrixes.

The HS-SPME technique is based on the distribution of target analyte in the headspace, sample matrix, and SPME fiber coating. Compared to other methods, SPME is a non-exhaustive extraction technique, which means only a small portion of target analytes can be extracted every time [16]. Previous studies have shown that flavor-enhanced oils contain hundreds of volatile compounds [1,24,25]. For example, Kim has detected 80 peaks from the volatiles of roasted perilla seed oil [24], Liu has observed 118 peaks, and identified 94 compounds from the volatiles of roasted peanut oil [1]. In the sealed vials, the distribution of volatiles is different in the three phases, which results in the selectivity of different volatiles by HS-SPME. On the other hand, non-degummed and undeodorized flavor oil is a complex sample matrix. The phospholipids, free fatty acids, and other compositions in oils will affect the volatilization and distribution of volatiles by binding, which is called the “matrix effect” [26]. For example, phenolic compounds were found to have an effect on the release of volatiles in virgin olive oil [27], which means the composition and property of the sample will affect the distribution of volatiles in the three phases. When there is a matrix effect, HS-SPME combined with traditional calibration methods, such as external standard and internal standard, has some limitations in the quantitation of volatiles in flavor oils. The stable isotope dilution analysis (SIDA) is a good approach that can effectively overcome the matrix effect [28]. However, the stable isotope standards are expensive and not all available commercially.

Multiple headspace solid-phase microextraction (MHS-SPME), which was developed by multiple headspace extraction (MHE) and firstly proposed by Suzuki and McAuliffe, along with Kolb [29,30,31], is one of the applications of exhaustive extraction by HS-SPME. The principle of the MHS-SPME procedure is based on the consecutive extraction of the same sample several times, while the total theoretical peak areas of the analyte corresponding to exhaustive extraction can be estimated from each individual extraction. The “matrix effect” can be eliminated by “exhaustive extraction”. Ezquerro et al. firstly applied MHS-SPME to quantify volatile organic compounds (VOCs) in multilayer food packaging materials in 2003 compared with the SA [32]. The results showed that MHS-SPME is a powerful method for the direct quantification of VOCs in solid samples. The comparison of the total peak areas of the calibration solution prepared with water and *n*-hexadecane showed that the peak area obtained by MHS-SPME were statistically equal, which proved that MHS-SPME could effectively eliminate the matrix effect [26]. Moreover, the use of MHS-SPME in wine flavor analysis proved that MHS-SPME could overcome the low repeatability when SPME was applied to the complex matrix [33]. To our knowledge, MHS-SPME combined with GC-MS has been successfully used to quantify volatile compounds in food products such as sausage, wine, herbal teas, roasted coffee, and so forth [33,34,35]. However, its application has not been performed in flavor-enhanced edible oils.

The aim of this study is to set up a novel and more accurate MHS-SPME-arrow-GC-MS method to quantify pyrazines in flavor-enhanced edible oils. This is the first time applying MHS-SPME-arrow to quantify flavor compounds in oils combined with internal standard (ISTD) while carried out in organic solvent for quantitative calibrations. There are three steps needed to develop the quantitative method based on MHS-SPME-arrow: (1) Optimizing the HS-SPME conditions for the maximum extraction efficiency and sensitivity improvement; (2) finding the appropriate sample loading suitable for MHS-SPME and clarifying the feasibility of SPME-arrow fiber in replacement of the traditional SPME fiber; and (3) validating the MHS-SPME-arrow method combined with the internal standard in solvent by selected conditions, in which the MHS-SPME-arrow and SIDA were compared using [^2^H_6_]-2-methyl-pyrazine.

## 2. Materials and Methods

### 2.1. Materials

Thirteen pyrazine standards (2-methyl-pyrazine, 2,5-dimethyl-pyrazine, 2,6-dimethyl-pyrazine, 2-ethyl-pyrazine, 2-ethyl-5-methyl-pyrazine, 2-ethyl-6-methyl-pyrazine, trimethyl-pyrazine, 2,6-diethyl-pyrazine, 3-ethyl-2,5-dimethyl-pyrazine, 2-ethyl-3,5-dimethyl-pyrazine, 2-methyl-3,5-diethyl-pyrazine, iso-propenyl-pyrazine, 5H-5-methyl-6,7-dihydrocyclopenta-pyrazine) and internal standard of 3-methyl-pyridine were purchased from Sigma-Aldrich Co. Ltd. (Shanghai, China). Isotopically labeled internal standard: [^2^H_6_]-2-methyl-pyrazine was purchased from CDN Isotopes Inc. (CDN, Canada). Caprylic capric triglyceride (ODO) was from Maitian Chemical Co. Ltd. (Shanghai, China).

Four SPME-arrow fibers, including polydimethylsiloxane (PDMS) (100 µm × 20 mm), polyacrylate (PA) (100 µm × 20 mm), polydimethylsiloxane/divinylbenzene (PDMS/DVB) (120 µm × 20 mm), polydimethylsiloxane/divinyl-benzene/carboxen (PDMS/DVB/CAR) (120 µm × 20 mm), and traditional SPME DVB/CAR/PDMS (50/30 µm × 10 mm) fibers were purchased from CTC Analytics AG (Zwingen, Switzerland). The holder of fibers was purchased from PAL System (Zwingen, Switzerland).

The refined pyrazine-free and flavor-enhanced rapeseed oils were obtained from the local supermarket, thus as the real samples of 3 flavor-enhanced oils, including peanut oil, sesame oil, and rapeseed oil. All oil samples were stored at 4 °C. Ethyl acetate was chromatographic grades.

### 2.2. Preparation of Standard Solutions

Standard solutions were prepared using ethyl acetate as the solvent. The combined stock solution of target analytes (13 pyrazines) and internal standard (3-methyl-pyridine) were prepared by weighing and storing in sealed amber glassware at 0 °C in the dark. The concentration of analytes (pyrazines and 3-methyl-pyridine) in stock solutions were approximately 1000 mg/L, and the standard calibration solution (approximately 25 mg/L) was prepared by diluting the stock solution accordingly.

### 2.3. Sample Preparation

A stock solution of the internal standard 3-methyl-pyridine (2000.0 mg/kg) was prepared by adding 20.0 mg of 3-methyl-pyridine to 10.0 g of ODO. Then, the stock solution of the internal standard was diluted to 50.0 mg/kg with ODO. The resulting solutions were stored at 0 °C in the dark.

The resulting internal standard solution (50.0 mg/kg) of 50.0 mg was added to 1.0 g of the oil sample, homogenized by vortex mixer afterward. The oil samples with internal standard (50.0 mg) were weighed into a 20 mL headspace vial sealed with a PTFE/silicone septum screw-cap. The vials were placed in the autosampler tray for HS-SPME analyses. The procedure for stable isotope dilution assay was the same as above.

### 2.4. MHS-SPME Conditions

For the HS-SPME method, the oil samples were pre-incubated at 80 °C for 20 min with the agitation speed of 450 rpm to release the volatile compounds prior to extraction, and then the fiber was exposed in the headspace of the vial at 50 °C for 50 min for equilibrium extraction. After the extraction of volatiles from the oil onto the fiber, the analytes were thermally desorbed from the fiber in the injector port of the chromatograph for 80 s and transferred to the chromatograph column where volatile compounds were separated. Finally, the analytes were identified and quantified by a mass spectrometer. After extraction and desorption, the SPME fiber was conditioned at 230 °C for 3 min. In the MHS-SPME method, the oil samples were taken 4 times at equal time intervals (of about 70 min).

### 2.5. GC–MS Conditions

A Combi PAL ingenious sample handling system (Ingenious Lab, Zwingen, Switzerland) used as an autosampler was mounted into the gas chromatograph. The change of liquid injector tool and SPME-arrow tool were the robotic steps for the analytical process. The gas chromatograph system was an Agilent 8890 coupled with a 5977B mass spectrometer (Agilent Technologies, Paolo Alto, CA, USA). A DB-FFAP analytical column (60 m × 0.25 mm, 0.25 µm) from Agilent Technologies was carried out to separate analytes. The SPME fiber device, which was used as an injector, was desorbed at high temperatures in the gas chromatography injector port to transfer analytes to gas chromatography device.

GC conditions: Fiber desorption time: 80 s; injector temperature: 230 °C; injection mode: Splitless; carrier gas: Helium (99.999% purity); flow rate: 1.0 mL/min. The oven temperature program was as follows: The initial temperature was 40 °C (1.5 min); the temperature was programmed from 40 to 100 °C (5 min) at 10 °C/min, from 100 to 150 °C (10 min) at 2 °C/min, from 150 to 185 °C at 5 °C/min, from 185 to 245 °C (8 min) at 20 °C/min, for a total running time of 65.5 min.

MSD conditions: MS was operated in EI mode (70 eV); acquisition was carried out in full scan and selected ion monitoring (SCAN&SIM) mode; and the selected ions were reported in Table 1. Other conditions include ion source temperature: 230 °C; quadrupole temperatures: 150 °C; and transfer line temperature: 280 °C. Data were collected and processed using MassHunter software (Agilent Technologies). Analytes were identified by retention time and selected ions, which are listed in Table 1.

### 2.6. Quantification of MHS-SPME

MHS-SPME is a process based on stepwise extraction of the analytes from the same sample, which can be seen as a combination of multiple headspace and headspace solid-phase microextraction [31,32]. After the same sample was consecutively extracted 4 times, the total peak area of analytes (A_T_) can be calculated by Equation (1):

(1) $$A_{T}=\sum_{i=1}^{i=1}A_{i}=\frac{A_{1}}{1-\beta }$$

where i is the number of extractions, A_i_ is the peak area of analytes in the ith extraction, A_1_ is the peak area of analytes in the first extraction, *β* is a constant between zero and one (0 ≤ *β* < 1), which can be calculated from Equation (2):

(2) $${\mathrm{lnA}}_{i}=(i-{1)\ln\beta +\mathrm{lnA}}_{1}$$

There is a linear relationship between ln A_i_ and i − 1, where ln *β* is the slope of the linear plot and can be calculated from a limited number (3 or 4) of extractions. Thus, the total amount of analytes in the system can be quantified by ISTD.

### 2.7. Calculation of ISTD

The detector response factors were calculated by the ethyl acetate solution of pyrazines and the internal standard. A liquid injection mode was used in this process by GC-MS. The response factors were calculated before samples were analyzed by MHS-SPME.

### 2.8. Statistical Analysis

Results were analyzed using ANOVA carried out using SPSS Statistical Software 18.0 (SPSS, Chicago, IL, USA), and the confidence interval was taken as 95%. All figures were generated using Origin 9.0, Adobe Illustrator, and Adobe Photoshop.

## 3. Results and Discussion

### 3.1. Optimization of HS-SPME Conditions

**Figure 1:**
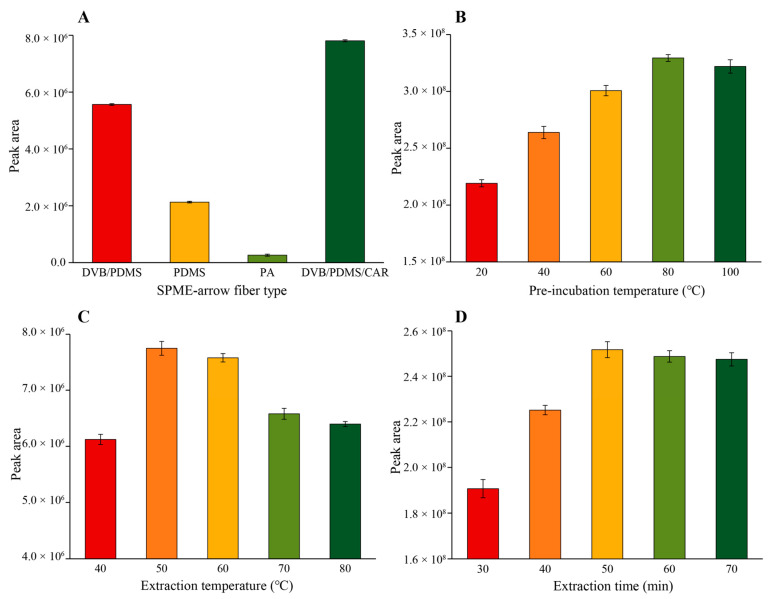
Influence of (**A**) the type of the fiber, (**B**) pre-incubation temperature, (**C**) extraction temperature, (**D**) extraction time on the total peak area of target compounds.

The experiments were performed on the flavor-enhanced rapeseed oil. Variables such as type of SPME-arrow fiber, pre-incubation temperature, extraction temperature, and time were studied to optimize the performance of HS-SPME. The HS-SPME conditions were optimized on the basis of peak areas of analytes and performed three times repeatedly. Figure 1 shows that the influence of the type of the fiber coating, pre-incubation temperature, extraction temperature, and time on the total peak area of target pyrazines.

The sensitivity and selectivity of the extraction method were determined by the properties of adsorbents on SPME fibers. The selection of a suitable fiber was the first step in developing the HS-SPME method. The performance of SPME fibers for the target compounds depends on the polarity of the analytes and the physical and chemical properties of coating types [16]. In this study, the extraction efficiency of the four commercial SPME fibers with different polar coatings was compared under the same conditions. The results showed significant differences among the various fiber coatings (*p* < 0.05) (Figure 1A). PDMS/DVB/CAR, which is produced with a cross-linked coating and contains bipolar coatings thus that can be used for the extraction of polar and non-polar VOCs, had the best performance (*p* < 0.05). The single-phase of PDMS (non-polar) and PA (polar) showed the poorest performance. These results were consistent with previous studies [36]. Therefore, a PDMS/DVB/CAR (120 µm × 20 mm) fiber was chosen for the optimization of the HS-SPME conditions and further used in this study.

After the type of coating was selected, the second important parameter was pre-incubation temperature. The role of the pre-incubation procedure is to volatilize the aroma compounds from the sample and build equilibrium between the sample and headspace. At the same pre-incubation time, the increase of temperature can promote the distribution of weak volatile components in the headspace and accelerate the building of equilibrium. However, higher temperatures may lead to the conversion and degradation of unstable substances. Five pre-incubation temperatures were studied: 20, 40, 60, 80, and 100 °C. The results (Figure 1B) showed that the higher pre-incubation temperature indeed contributed to the volatilization and equilibrium of pyrazines. However, the total peak area was not significantly different between 80 °C and 100 °C. Thus, 80 °C for pre-incubation temperature was chosen as the SPME condition for further studies.

Extraction temperature is an important factor affecting the extraction efficiency of aroma compounds. The increasing temperature is conducive to the release of aroma compounds from the matrix at the same extraction time. However, high temperatures may also cause the degradation of components in the food matrix and decrease the absorbent ability of SPME fibers. Five extraction temperatures were studied: 40, 50, 60, 70, and 80 °C. According to Figure 1C, the extraction temperature of 50 °C was selected.

The effect of different extraction times (30, 40, 50, 60, and 70 min) was also evaluated at 50 °C. The optimum time was required to reach equilibrium in three phases: The fiber coating, the headspace, and the sample. SPME under equilibrium is an important condition for carrying out the MHS-SPME operation. Compared with extraction without equilibrium, extraction under equilibrium has better repeatability [32]. As shown in Figure 1D, 50 min was chosen as the optimal extraction time, which ensured extraction efficiency and established the equilibrium of pyrazines in three phases.

On that basis of the above experiments, the PDMS/DVB/CAR (120 µm × 20 mm) fiber coating was chosen, and the optimal HS-SPME conditions were pre-incubation temperature: 80 °C; extraction temperature: 50 °C; and extraction time: 50 min.

### 3.2. Amount of Oil Sample

The basic operation of MHS-SPME involves repeating extraction several times from the same sample, while the peak area decreases exponentially with the number of extractions. Therefore, the amounts of the sample should not only meet the LOQs but also ensure the linearity between ln A_i_ versus the extraction numbers (i − 1), where ln A_i_ shows linear decay with the number of extractions (Equation (2)). In order to satisfy that requirement, 0.4 < *β* < 0.95 should be fulfilled, which indicates the slope of the linear plot ln A_i_ versus i−1 must less than −0.0513 and more than −0.9163 [37]. Compared with the traditional SPME fiber, the SPME-arrow device has a larger sorbent phase and showed higher sensitivities and extraction amount [20]. In this study, the effects of seven sample amounts and two fiber types on *β* and coefficient of determination (R^2^) were studied on flavor-enhanced rapeseed oils using the 20 mL HS vial.

**Figure 2:**
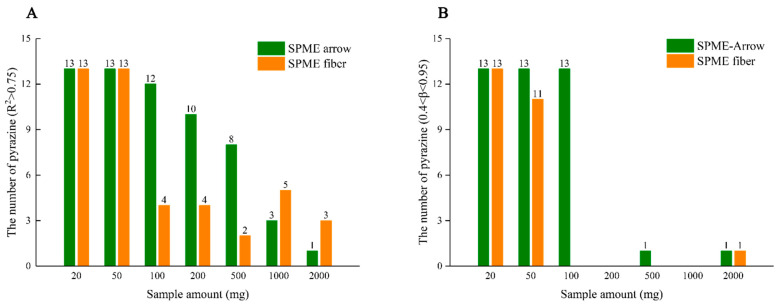
The number of pyrazines that met the following conditions: (**A**) R^2^ > 0.75; (**B**) 0.4 < *β* < 0.95, using solid phase microextraction (SPME)-arrow fiber and traditional SPME fiber, respectively.

For MHS-SPME, two conditions should be met: (1) R^2^ > 0.75, (2) 0.4 < *β* < 0.95, indicating that the ln A_i_ was linearly decayed with the number of extractions. Figure 2A shows the sample amount meeting R^2^ > 0.75 while Figure 2B meeting 0.4 < *β* < 0.95. As shown in Figure 2, traditional SPME fiber exhibited the most pyrazines that met requirements when the sample amount was 20.0 mg, while the SPME-arrow fiber showed the highest number at 20.0 mg and 50.0 mg samples. This was in line with the advantage of the SPME-arrow fiber that larger sorbent phase volume gives higher extraction capacity. When the sample amount was greater than 200 mg, almost no *β* satisfied the MHS-SPME applicable requirement, regardless if the traditional SPME fiber or SPME–arrow fiber was used. The reason might be that the headspace was saturated in the multiple headspace extractions. However, the low sample amount may lead to poor repeatability due to the mass of analytes closed to LOQs, which means that the SPME–arrow fiber was more suitable for the quantitation of pyrazines in flavor oils by MHS-SPME. Therefore, 50.0 mg was selected as the sample mass in the following study.

### 3.3. Validation of MHS-SPME-Arrow Method

The analytical performance of the MHS-SPME-arrow-GC-MS was evaluated in terms of LOD, LOQ, inter-day precision, intra-day precision, and recovery, as shown in Table 1 and Table 2.

**Table 1:** List of retention time, selected ions, limits of detection (LOD), limits of quantification (LOQ), inter-day precision, intra-day precision of 13 target pyrazines in oils.

Compound	Retention Time (min)	Selected Ions ^1^	LOD ^2^ (ng/g)	LOQ ^2^ (ng/g)	Intra-Precision RSD (%)	Inter-Precision RSD (%)	Concentration Level ^3^ (ng/g)
3-methyl, pyridine ^4^	15.93	66, 92, **93**	--	--	--	--	--
2-methyl-pyrazine	14.78	67, 53, **94**	3	6	7.13	8.66	907
2,5-dimethyl-pyrazine	16.62	42, 81, **108**	5	20	5.11	5.67	5011
2,6-dimethyl-pyrazine	16.90	40, 42, **108**	8	20	13.68	12.14	264
Ethyl-pyrazine	17.05	80, 108, **107**	2	6	12.28	14.77	192
2-ethyl-6-methyl-pyrazine	18.93	94,122, **121**	3	6	9.64	12.27	363
2-ethyl-5-methyl-pyrazine	19.21	94, 122, **121**	3	10	7.83	9.56	586
Trimethyl-pyrazine	19.80	42, 81, **122**	3	6	10.61	11.01	622
2,6-diethyl-pyrazine	21.04	108, 136, **135**	3	10	13.69	15.10	43
3-ethyl-2,5-dimethyl-pyrazine	21.41	108, 136, **135**	4	20	11.28	10.78	1837
2-ethyl-3,5-dimethyl-pyrazine	22.28	108, 136, **135**	5	20	13.21	15.08	129
3,5-diethyl-2-methyl-pyrazine	23.63	122, 135, **149**	6	20	13.09	12.99	177
Isopropenyl-pyrazine	29.02	67, 120, **119**	60	180	12.67	14.98	190
5H-5-Methyl-6,7-dihydrocyclopenta-pyrazine	30.18	119, 133, **134**	25	50	10.00	14.02	94

^1^ Quantification ions are present in bold. ^2^ LOD and LOQ were obtained by standard solution of pyrazines, and precision was evaluated by commercial flavor rapeseed oil. ^3^ The concentration of pyrazines in the sample during the repeatability test. ^4^ Internal standards. Abbreviations: RSD—relative standard deviation.

This method allows the analysis of a variety of different pyrazines, and the selected internal standard is pyridine with a similar structure to pyrazines. Therefore, good selectivity is a necessary requirement for the correct identification and quantification of all analytes. The identification and quantitation of target compounds, including internal standard, were based on the retention time and different selected ions, which are listed in Table 1.

For the LOD and LOQ determinations, the refined rapeseed oil (“pyrazine-free”) was used as the matrix to prepare standard solutions. The suitable concentration was established as LOD and LOQ of pyrazines with the signal-to-noise ratio (S/N) of pyrazines being 3.0 and 10.0, respectively [38]. As shown in Table 1, the LODs and LOQs of 13 pyrazines were in the range of 2–60 ng/g and 6–180 ng/g, respectively, indicating that MHS-SPME can be used in routine quantitation of pyrazines.

To evaluate the precision of the method, five parallel experiments were carried out on commercial flavor-enhanced rapeseed oils to calculate the intra-day precision. The inter-day precision was determined by analyzing the same sample five times a week for three weeks. Both intra-day precision and inter-day precision were expressed as the relative standard deviation (RSD). The results showed that the RSD of intra-day and inter-day were both lower than 16%, verifying the good precision of the MHS-SPME-arrow method. Model experiments were also carried out to evaluate accuracy.

Three spiked concentration levels in refined rapeseed oil were also studied by the standard addition method (SA). The results in Table 2 showed that the recoveries of pyrazines analyzed were in the range of 90% to 115% in all cases, indicating that the method was reliable and accurate within the concentration range of the model experiments.

**Table 2:** Recoveries of 13 pyrazines in refined rapeseed oil at different concentration levels.

Compound	Spiked Level (ng/g)	Recovery ^1^ (%)	Spiked Level (ng/g)	Recovery ^1^ (%)	Spiked Level (ng/g)	Recovery ^1^ (%)
2-methyl-pyrazine	342	104.4 ± 15.0	1710	106.1 ± 4.13	3420	98.5 ± 6.9
2,5-dimethyl-pyrazine	642	99.8 ± 7.7	3210	111.3 ± 13.0	6420	93.9 ± 12.5
2,6-dimethyl-pyrazine	382	109.8 ± 6.0	1910	104.3 ± 4.2	3820	100.9 ± 13.7
Ethyl-pyrazine	364	105.2 ± 2.9	1820	95.3 ± 9.5	3640	97.1 ± 14.4
2-ethyl-6-methyl-pyrazine	330	104.8 ± 8.4	1652	105.5 ± 6.5	3305	94.2 ± 10.7
2-ethyl-5-methyl-pyrazine	85	97.8 ± 8.3	425	93.4 ± 7.9	851	113.6 ± 5.5
Trimethyl-pyrazine	336	112.9 ± 3.1	1680	109.7 ± 5.6	3360	97.9 ± 10.6
2,6-diethyl-pyrazine	256	96.1 ± 6.5	1280	107.8 ± 8.1	2560	115.4 ± 7.2
3-ethyl-2,5-dimethyl-pyrazine	252	102.2 ± 6.7	1260	112.3 ± 15.7	2520	106.7 ± 7.9
2-ethyl-3,5-dimethyl-pyrazine	314	112.0 ± 14.0	1570	106.7 ± 6.3	3140	91.9 ± 9.4
3,5-diethyl-2-methyl-pyrazine	284	105.3 ± 10.3	1420	109.1 ± 6.23	2840	110.5 ± 6.5
Isopropenyl-pyrazine	308	98.7 ± 7.1	1540	95.34 ± 9.1	3080	93.0 ± 10.2
5H-5-Methyl-6,7-dihydrocyclopenta-pyrazine	98	96.5 ± 5.1	490	109.2 ± 6.7	980	111.7 ± 11.0

^1^ Recovery calculated from three concentration and three replicates.

The stable isotope dilution analysis (SIDA) has proven to be very precise in the model experiments, even at a very low extraction yield [39]. The main reason is that SIDA uses the most suitable internal standard: Stable isotopes of the analytes, which can fully be recovered for the losses. This study compared the quantitative results of SIDA and MHS-SPME of flavor-enhanced rapeseed oil, using 2-methyl-pyrazine labeled ^2^H_6_ as the stable isotope internal standard. The concentration of 2-methyl-pyrazine was 1.21 ± 0.13 µg/g for SIDA and 1.43 ± 0.11 µg/g for MHS-SPME-arrow, respectively. There was no difference (*p* >0.05) by statistical analysis. Thus, the data showed that the MHS-SPME-arrow could be applied to the analysis of these pyrazines in oil samples. By this conclusion, the method has been set up after condition optimization and SIDA method verification. An efficient and effective MHS-SPME-arrow-GCMS analytical protocol is established and can be used to quantify the flavor compounds in oils.

### 3.4. Analysis of Real Samples

**Figure 3:**
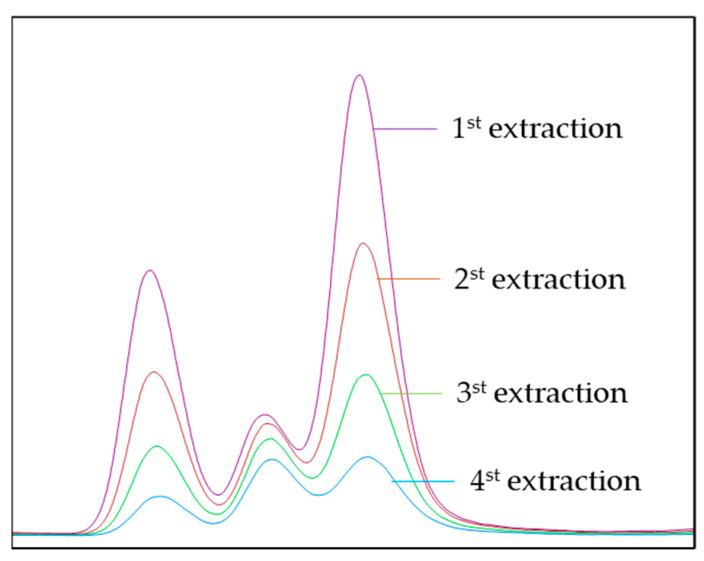
Chromatogram of 2-ethyl-5-methyl-pyrazine of four successive HS-SPME extractions from flavor-enhanced peanut oil.

Flavor-enhanced oils are usually produced by the traditional pressing process, mainly including roasting, pressing, and filtering. Heterocyclic volatiles are commonly produced by the Maillard reactions among proteins, amino acids, and sugars during oilseed roasting [40]. Pyrazines are one group of the main Maillard reaction products and also give the main source of roasting-like aroma in flavor oils [6]. Flavor-enhanced sesame and peanut oils are the main representatives of such oils, while rapeseed oil also has a good market in China for its unique spicy and roasted flavor, occupying more than 30% of the entire Chinese rapeseed oil market [41]. For the quantification of pyrazines in the three market product samples, MHS-SPME-arrow-GC-MS were carried out as set up above. Prior to analysis of the samples, the standard calibration solution of pyrazines and 3-methyl-pyridine with known concentrations were analyzed to calibrate the detector responses. Taking 2-ethyl-5-methyl-pyrazine in flavor-enhanced peanut oil as an example, Figure 3 showed the change of peak areas for four successive extractions. An exponential decrease in the target compound with the number of extractions was observed. The average concentrations and standard deviations of pyrazines together with R^2^ and slope of the linear plot ln A_i_ versus i − 1, were shown in Table 3.

Thirteen pyrazines were found in three oils. According to the discussion in Section 3.2, the optimal sample amount was taken as 50.0 mg. To apply the MHS-SPME method, the following requirements must be met: R^2^ > 0.75 and −0.9163 < slope < −0.0513. The results showed that the R^2^ of pyrazines in three samples was greater than 0.98, of which more than 90% was bigger than 0.99. All the slopes were in the range of −0.9163~−0.0513. It was shown that the ln A_i_ decreased linearly with the number of extractions, and the linear relationship was good, fitting to the MHS-SPME-arrow method.

There were significant differences in the concentrations of pyrazines in different types of oils (*p* < 0.05). The total concentrations of pyrazines in the three samples were, in order from high to low: Sesame oil, peanut oil, and rapeseed oil. The total amount of pyrazines in sesame oil was close to 14 times that of rapeseed oil. The pyrazines with the highest concentration were 2-methyl-pyrazine and 2,5-dimethyl-pyrazine in three oils. As described by previous studies [1,11], pyrazines may be the best indicator to measure roasted flavor intensity. It is now clear also that sesame oil has the strongest roasted flavor as the concentration of pyrazines is the highest.

## 4. Conclusions

In summary, a reliable MHS-SPME-arrow-GC-MS method combined with ISTD for the quantitation of 13 pyrazines in flavor oils was developed. The SPME-arrow fiber was found to be more suitable for the MHS-SPME method, and the PDMS/DVB/CAR fiber was selected. The highest efficiency was performed under selected HS-SPME conditions. The novel method was verified with the stable isotope dilution analysis method and showed high sensitivity and accuracy. Additionally, the pyrazines of three market product samples were analyzed and quantified using the new method, which proved that the MHS-SPME-arrow-GC-MS was suitable to quantify pyrazines in oils. In the future development, this method has great opportunities in the quantitation of other aroma active compounds such as alcohols and pyrroles. At the same time, as an absolute quantitative method to eliminate the matrix effect, this method also has an obvious limitation. That is that it requires multiple extractions for a single analysis, which means it takes a much longer time than the conventional HS-SPME-GC-MS. Therefore, there are some aspects for further improvement for this newly proposed method.

## Figures and Tables

**Table 3:** The coefficient of determination (R^2^) and slope of the linear plot ln A_i_ versus i − 1, mean concentration, and standard deviation of pyrazines in peanut, sesame, and rapeseed oils.

	Peanut Oil			Sesame Oil			Rapeseed Oil		
	Concentration (mg/kg)	R^2^	Slope	Concentration (mg/kg)	R^2^	Slope	Concentration (mg/kg)	R^2^	Slope
2-methyl-pyrazine	2.87 ± 0.18	0.9962	−0.71	15.14 ± 2.46	0.9943	−0.47	0.58 ± 0.00 ^1^	0.9948	−0.56
2,5-dimethyl-pyrazine	3.23 ± 0.21	0.9990	−0.75	9.50 ± 1.01	0.9945	−0.56	1.07 ± 0.00	0.9979	−0.73
2,6-dimethyl-pyrazine	1.10 ± 0.07	0.9974	−0.74	3.98 ± 0.41	0.9937	−0.54	0.30 ± 0.02	0.9991	−0.65
Ethyl-pyrazine	0.38 ± 0.02	0.9973	−0.73	1.74 ± 0.12	0.9930	−0.49	0.09 ± 0.00	0.9932	−0.61
2-ethyl-6-methyl-pyrazine	0.76 ± 0.06	0.9998	−0.52	3.62 ± 0.71	0.9980	−0.41	0.24 ± 0.00	0.9991	−0.50
2-ethyl-5-methyl-pyrazine	0.96 ± 0.07	0.9999	−0.47	2.21 ± 0.46	0.9986	−0.41	0.21 ± 0.00	0.9988	−0.48
Trimethyl-pyrazine	1.07 ± 0.09	1.0000	−0.48	5.49 ± 1.16	0.9989	−0.38	0.29 ± 0.00	0.9995	−0.46
2,6-diethyl-pyrazine	0.06 ± 0.01	1.0000	−0.34	0.26 ± 0.06	0.9996	−0.27	0.02 ± 0.00	0.9997	−0.32
3-ethyl-2,5-dimethyl-pyrazine	0.83 ± 0.07	0.9998	−0.31	5.08 ± 1.22	0.9996	−0.25	0.55 ± 0.00	0.9999	−0.29
2-ethyl-3,5-dimethyl-pyrazine	0.12 ± 0.01	0.9997	−0.29	0.54 ± 0.13	0.9991	−0.24	0.03 ± 0.00	0.9897	−0.28
3,5-diethyl-2-methyl-pyrazine	0.05 ± 0.01	0.9965	−0.19	0.23 ± 0.05	0.9970	−0.16	0.02 ± 0.00	0.9959	−0.17
Isopropenyl-pyrazine	ND ^2^	-- ^3^	--	0.93 ± 0.22	0.9994	−0.27	ND	--	--
5H-5-Methyl-6,7-dihydrocyclopenta-pyrazine	0.06 ± 0.01	0.9964	−0.21	0.47 ± 0.10	0.9862	−0.18	ND	--	--
Total pyrazines	11.51 ± 0.78			49.18 ± 3.25			3.42 ± 0.03		

^1^ Standard deviation less than 0.005 was considered as 0.00. ^2^ ND, not detected. ^3^ Slope and R^2^ not shown as it showed no linear decay.

## Data Availability

Data are contained within the article.

## References

[1] Liu X., Jin Q., Liu Y., Huang J., Wang X., Mao W., Wang S. (2011). Changes in Volatile Compounds of Peanut Oil during the Roasting Process for Production of Aromatic Roasted Peanut Oil. J. Food Sci..

[2] Guerra P.V., Yaylayan V.A. (2010). Dimerization of Azomethine Ylides: An Alternate Route to Pyrazine Formation in the Maillard Reaction. J. Agric. Food Chem..

[3] An A., Kimpe N.D. (2009). Formation of pyrazines from ascorbic acid and amino acids under dry-roasting conditions—ScienceDirect. Food Chem..

[4] Kwon T.Y., Park J.S., Jung M.Y. (2013). Headspace–Solid Phase Microextraction–Gas Chromatography–Tandem Mass Spectrometry (HS-SPME-GC-MS^2^) Method for the Determination of Pyrazines in Perilla Seed Oils: Impact of Roasting on the Pyrazines in Perilla Seed Oils. J. Agric. Food Chem..

[5] Gama A.P., Adhikari K. (2019). Sensory Characterization of Dominant Malawi Peanut Varieties After Roasting. J. Food Sci..

[6] Jia X., Zhou Q., Wang J., Liu C., Huang F., Huang Y. (2019). Identification of key aroma-active compounds in sesame oil from microwaved seeds using E-nose and HS-SPME-GC×GC-TOF/MS. J. Food Biochem..

[7] Qu F., Zhu X., Ai Z., Ai Y., Qiu F., Ni D. (2019). Effect of different drying methods on the sensory quality and chemical components of black tea. LWT Food Sci. Technol..

[8] Baker G.L., Cornell J.A., Gorbet D.W., O’Keefe S.F., Talcott S.T. (2010). Determination of Pyrazine and Flavor Variations in Peanut Genotypes During Roasting. J. Food Sci..

[9] Shimoda M., Shiratsuchi H., Nakada Y., Yin W., Osajima Y. (1996). Identification and Sensory Characterization of Volatile Flavor Compounds in Sesame Seed Oil. J. Agric. Food Chem..

[10] Poehlmann S., Schieberle P. (2013). Characterization of the Aroma Signature of Styrian Pumpkin Seed Oil (Cucurbita pepo subsp. pepo var. Styriaca) by Molecular Sensory Science. J. Agric. Food Chem..

[11] Zhou Q., Xiao J., Yao Y., Wang B., Huang F. (2019). Characterization of the Aroma-Active Compounds in Commercial Fragrant Rapeseed Oils via Monolithic Material Sorptive Extraction. J. Agric. Food Chem..

[12] Kanavouras A., Kiritsakis A., Hernandez R.J. (2005). Comparative study on volatile analysis of extra virgin olive oil by dynamic headspace and solid phase micro-extraction. Food Chem..

[13] David F., Pawliszyn J. (2012). 4.21—Application of Stir-Bar Sorptive Extraction in Food Analysis. Comprehensive Sampling and Sample Preparation.

[14] Cecchi T., Alfei B. (2013). Volatile profiles of Italian monovarietal extra virgin olive oils via HS-SPME-GC-MS: Newly identified compounds, flavors molecular markers, and terpenic profile. Food Chem..

[15] Matheis K., Granvogl M. (2016). Characterization of Key Odorants Causing a Fusty/Musty Off-Flavor in Native Cold-Pressed Rapeseed Oil by Means of the Sensomics Approach. J. Agric. Food Chem..

[16] Pawliszyn J. (2012). Handbook of Solid Phase Microextraction.

[17] Pasqualone A., Paradiso V.M., Summo C., Caponio F., Gomes T. (2014). Influence of Drying Conditions on Volatile Compounds of Pasta. Food Bioprocess Technol..

[18] Giannone V., Giarnetti M., Spina A., Todaro A., Pecorino B., Summo C., Caponio F., Paradiso V.M., Pasqualone A. (2018). Physico-chemical properties and sensory profile of durum wheat Dittaino PDO (Protected Designation of Origin) bread and quality of re-milled semolina used for its production. Food Chem..

[19] Liu X., Wang S., Tamogami S., Chen J., Zhang H. (2021). Volatile Profile and Flavor Characteristics of Ten Edible Oils. Anal. Lett..

[20] Song N.-E., Lee J.-Y., Lee Y.-Y., Park J.-D., Jang H.W. (2019). Comparison of headspace–SPME and SPME-Arrow–GC–MS methods for the determination of volatile compounds in Korean salt–fermented fish sauce. Appl. Biol. Chem..

[21] Nam T.G., Lee J.Y., Kim B.K., Song N.E., Jang H.W. (2019). Analyzing volatiles in brown rice vinegar by headspace solid-phase microextraction (SPME)–Arrow: Optimizing the extraction conditions and comparisons with conventional SPME. Int. J. Food Prop..

[22] Lee J.-Y., Kim W.S., Lee Y.-Y., Choi Y.-S., Choi H., Jang H.W. (2019). Solid-phase microextraction Arrow for the volatile organic compounds in soy sauce. J. Sep. Sci..

[23] Manousi N., Rosenberg E., Zachariadis G.A. (2020). Solid-Phase Microextraction Arrow for the Sampling of Volatile Organic Compounds in Milk Samples. Separations.

[24] Kim S.J., Yoon H.N., Rhee J.S. (2000). The effects of roasting temperatures on the formation of headspace volatile compounds in perilla seed oil. J. Am. Oil Chem. Soc..

[25] Zhang W., Rui W., Yuan Y., Yang T., Liu S. (2016). Changes in volatiles of palm kernel oil before and after kernel roasting. LWT Food Sci. Technol..

[26] Ezquerro Ó., Pons B., Tena M.A.T. (2003). Evaluation of multiple solid-phase microextraction as a technique to remove the matrix effect in packaging analysis for determination of volatile organic compounds. J. Chromatogr. A.

[27] Genovese A., Yang N., Linforth R., Sacchi R., Fisk I. (2018). The role of phenolic compounds on olive oil aroma release. Food Res. Int..

[28] Pickard S., Becker I., Merz K.H., Richling E. (2013). Determination of the alkylpyrazine composition of coffee using stable isotope dilution-gas chromatography-mass spectrometry (SIDA-GC-MS). J. Agric. Food Chem..

[29] Suzuki M., Tsuge S., Takeuchi T. (1970). Gas chromatographic estimation of occluded solvents in adhesive tape by periodic introduction method. Anal. Chem..

[30] Mcauliffe C.D. (1971). GC Determination of Solutes by Multiple Phase Equilibration. Chem. Technol..

[31] Kolb B. (1982). Multiple headspace extraction—A procedure for eliminating the influence of the sample matrix in quantitative headspace, gas chromatography. Chromatographia.

[32] Ezquerro Ó., Pons B., Tena M.A.T. (2003). Direct quantitation of volatile organic compounds in packaging materials by headspace solid-phase microextraction–gas chromatography–mass spectrometry. J. Chromatogr. A.

[33] Gómez-Ariza J.L., García-Barrera T., Lorenzo F., Beltrán R. (2006). Use of multiple headspace solid-phase microextraction and pervaporation for the determination of off-flavours in wine. J. Chromatogr. A.

[34] Canellas E., Vera P., Nerin C. (2016). Multiple headspace-solid phase microextraction for the determination of migrants coming from a self-stick label in fresh sausage. Food Chem..

[35] Sgorbini B., Cagliero C., Acquadro S., Marengo A., Cordero C., Liberto E., Bicchi C., Rubiolo P. (2019). Evaluation of volatile bioactive secondary metabolites transfer from medicinal and aromatic plants to herbal teas: Comparison of different methods for the determination of transfer rate and human intake. J. Chromatogr. A.

[36] Liu G., Xu S., Wang X., Jin Q., Xu X., Shen Y., Xu G., Zhang H. (2016). Analysis of the volatile components of tea seed oil (Camellia sinensis O. Ktze) from China using HS-SPME-GC/MS. Int. J. Food Sci. Technol..

[37] Tena M.T., Carrillo J.D. (2007). Multiple solid-phase microextraction: Theory and applications. TrAC Trends Anal. Chem..

[38] Martínez-Uruuela A., González-Sáiz J., Pizarro C. (2004). Optimisation of the derivatisation reaction and subsequent headspace solid-phase microextraction method for the direct determination of chlorophenols in red wine. J. Chromatogr. A.

[39] Schieberle P., Grosch W. (1987). Evaluation of the flavour of wheat and rye bread crusts by aroma extract dilution analysis. Z. Lebensm. Unters. Forsch..

[40] Van Lancker F., Adams A., De Kimpe N. (2010). Formation of pyrazines in Maillard model systems of lysine-containing dipeptides. J. Agric. Food Chem..

[41] Zhang Y., Zhai X., Gao L., Jin J., Zhong Q., Sun C., Yan L., Liu R., Akoh C.C., Jin Q. (2017). Quality of Wood-Pressed Rapeseed Oil. J. Am. Oil Chem. Soc..

